# Systemic Lupus Erythematosus and Thyroid Autoimmunity

**DOI:** 10.3389/fendo.2017.00138

**Published:** 2017-06-19

**Authors:** Silvia Martina Ferrari, Giusy Elia, Camilla Virili, Marco Centanni, Alessandro Antonelli, Poupak Fallahi

**Affiliations:** ^1^Department of Clinical and Experimental Medicine, University of Pisa, Pisa, Italy; ^2^Department of Medico-Surgical Sciences and Biotechnologies, “Sapienza” University of Rome, Latina, Italy

**Keywords:** systemic lupus erythematosus, autoimmune thyroiditis, hypothyroidism, Graves’ disease, thyroid cancer, AbTPO, AbTg, CXCL10

## Abstract

Most of the studies present in the literature show a high prevalence, and incidence, of new cases of hypothyroidism and autoimmune thyroiditis (AT) in systemic lupus erythematosus (SLE) patients, overall in female gender. A limited number of cases of Graves’ disease have been also reported in SLE patients, in agreement with the higher prevalence of thyroid autoimmunity. It has been also demonstrated that a Th1 predominance is associated with AT in SLE patients. Furthermore, a higher prevalence of papillary thyroid cancer has been recently reported in SLE, in particular in the presence of thyroid autoimmunity. However, studies in larger number of SLE patients are needed to confirm findings about thyroid cancer. On the whole, data from literature strongly suggest that female SLE patients, with a high risk (a normal but at the higher limit thyroid-stimulating hormone value, positive antithyroid peroxidase antibodies, a hypoechoic pattern, and small thyroid), should undergo periodic thyroid function follow-up, and appropriate treatments when needed. A careful thyroid monitoring would be opportune during the follow-up of these patients.

## Introduction

Systemic lupus erythematosus (SLE) is an autoimmune disease affecting mostly joints, skin, blood vessels, heart, lungs, kidneys, liver, and nervous system, in which the immune system attacks tissues and cells leading to inflammation and damage (1). The course of SLE is variable, with periods of exacerbation (called flares) alternating with periods of remission. SLE occurs nine times more often in the female gender than in male, and it is more frequent in people of non-European descent (1). Different types of autoantibodies are present in SLE patients (2). The difference of clinical features of SLE in different patients is due to the complexity of the risk factors (genetic, hormonal, and environmental), and the variety of circulating autoantibodies present (2, 3). Because of the presence of many different autoantibodies, SLE is classified as a “B-cell disease.” Circulating blood lymphocytes in SLE patients show a Th2-like profile (4); however, Th1 lymphocytes and interferon (IFN)-γ have been demonstrated to be important for the immune pathogenesis of SLE (5). In fact, it has been shown that the assistance of the Th1 lymphocytes is necessary for the development of the disease, and in the absence of a functioning Th1 immune response “helpless” B cells are not functional enough to trigger SLE inflammation (6). Circulating autoantibodies, immune complex deposition, and complement activation are SLE hallmarks; however, a growing body of evidence has shown the importance of cytokines/chemokines in this disease, such as interleukin (IL)-6, B lymphocyte stimulator, IL-17, type I IFNs, tumor necrosis factor (TNF)-α, and Th1 chemokines. These cytokines/chemokines are important in the orchestration, maturation, differentiation, and activation of various immune competent cells, which mediate the local inflammation and produce the tissue injury.

Systemic lupus erythematosus is associated with many other autoimmune diseases, and autoimmune thyroid disorders (AITD) too (7, 8).

Here, we review the scientific literature about the association of SLE and AITD.

## The Association of SLE and AITD

After the initial case reports describing a coexistence of SLE and AITD, many systematic studies have evaluated the possible association of SLE and AITD (Table 1).

**Table 1:** Prevalence of thyroid autoimmunity in SLE patients versus controls, in the studies that have an internal control matched by gender and age.

Reference	SLE patients (*n*)	AITD% in SLE patients	Controls (*n*)	AITD% in controls	*P*
Antonelli et al. (7)	213	Thyroid autoimmunity 34.7%	426	Thyroid autoimmunity 15.1%	<0.001
Antonelli et al. (8)	153	AbTg or AbTPO positivity 33%; AbTg and AbTPO positivity 12%	459 iodine-deficient controls and 459 iodine-sufficient controls	AbTg or AbTPO positivity 11% in iodine-deficient controls; AbTg or AbTPO positivity 13% in iodine-sufficient controls; AbTg and AbTPO positivity 2% in iodine-deficient controls; AbTg and AbTPO positivity 3% in iodine-sufficient controls	<0.0001 for AbTg or AbTPO positivity; 0.001 for AbTg and AbTPO positivity
Weetman and Walport (14)	41	ThyAb 51%	41	ThyAb 27%	<0.05
Vianna et al. (17)	100	ThyAb 21%; AbTg 11%	100	ThyAb 16%; AbTg 2%	0.009 for AbTg
Mihailova et al. (23)	12 children with SLE	AbTg 58%	27 children having juvenile chronic arthritis	AbTg 63% autoimmune thyroiditis 44.4%	Not reported
Shahin et al. (24)	45; AbTg and AbM were assessed in 27 patients	AbTg 18.5%	20	0	ns
Mader et al. (27)	77	AbTg 7.8%; AbTPO 5.2%	52	AbTg 7.7%; AbTPO 7.7%	ns
Appenzeller et al. (28)	524	Symptomatic AITD was observed in 6.1%	50	Symptomatic AITD was observed in 2%	>0.05
Lazúrová et al. (29)	80 patients with SLE or RA (12 SLE and 68 RA)	Prevalence of AITD 24%	34	Prevalence of AITD 8%	<0.05
Kumar et al. (30)	100	Prevalence of ThyAb 30%	100	Prevalence of ThyAb 10%	<0.05
Lin et al. (33)	1,633	Cumulative incidence of thyroid disease 8.1%	6,532	Cumulative incidence of thyroid disease 16.9%	<0.001

*AbM, antimicrosomal antibodies; AITD, autoimmune thyroid disorders; AbTg, anti-thyroglobulin antibodies; AbTPO, anti-thyroperoxidase antibodies; RA, rheumatoid arthritis; SLE, systemic lupus erythematosus; ThyAb, thyroid autoantibodies*.

### Prevalence of Thyroid Autoantibodies (ThyAb) in SLE Patients

Serological overlap among SLE, rheumatoid arthritis (RA), and AITD exists (9–12).

A first study evaluated thyroid disorders in 319 SLE patients showing 9 with thyrotoxicosis, 3 with hypothyroidism, and 2 with thyroiditis, suggesting a higher prevalence of thyroid disorders in SLE patients (13). Weetman and Walport compared the prevalence of ThyAb and abnormal thyroid-stimulating hormone (TSH) levels in 41 SLE patients, versus age- and sex-matched controls. A significant higher prevalence of ThyAb (51%) was observed in SLE compared to (27%) controls. Furthermore, hypothyroidism was observed in 10 SLE patients and 5 controls, usually in association with circulating ThyAb (14). In another study, 18% of SLE patients had positive antimicrosomal antibodies (AbM). Euthyroid sick syndrome (ESS) was diagnosed in 15% of SLE patients, and true or initial primary hypothyroidism in 5, and 39%, respectively. ThyAb were present in 45% of SLE patients with high TSH (15). Anti-TSH receptor antibodies were also evaluated in 28 SLE patients with thyroid disorders. 10/28 patients demonstrated thyroid-stimulating immunoglobulins (TSI) activity, while 5 patients had evidence of TSH-binding inhibitor immunoglobulin (TBII) activity. The TSI, or TBII activity, was not associated with the abnormal thyroid function tests (16). Vianna et al. studied 100 SLE patients for the presence of ThyAb and thyroid disease. The ThyAb were similar in SLE (21%) and controls (16%); AbTg were present in 11% SLE and only 2% controls; AbM levels were also different (median levels: SLE = 400; controls = 100). A higher frequency of clinical thyroid disease was observed in SLE patients with ThyAb (5/21; 3 hypothyroid, 2 hyperthyroid) than in those without (1/79). SLE patients with ThyAb were significantly older (mean age 47.5 years) than those without (mean age 37.5 years) (17). In a group of SLE female patients, thyroxine (T4) was significantly lower than in controls and 45.5% of patients with active SLE presented ThyAb. TSH basal, and after TRH stimulation, were significantly higher in patients with active SLE. ThyAb were not found in patients with inactive SLE (18). In 45 Chinese SLE patients, 24 (53.3%) had altered echographic findings. AbM and/or AbTg were reported in 21 patients (46.7%), and TBII in only one. Ten patients (22.2%) showed an altered thyroid function. Hashimoto’s thyroiditis was reported in four patients (8.8%), two of whom had hypothyroidism. The mean disease duration was longer in patients with thyroid anomalies (*P* < 0.05) (19). Among 129 SLE patients from Singapore, 8.9% had hyperthyroidism, 3.9% Hashimoto’s thyroiditis, and 47.8% ESS. AbM or AbTg were present in 32.2% (20). Kausman and Isenberg evaluated 150 SLE patients, showing that 31 (21%) were ThyAb positive. Follow-up data were available on 20 ThyAb positive patients (average of 7.9 years), of whom 12 (60%) were persistently ThyAb positive, while 8 (40%) were negative on at least one occasion during the follow-up. Five cases of clinical thyroid disease that were diagnosed, and 2/3 cases of subclinical elevation of TSH, occurred in the group with persistently positive ThyAb. Four (9%)/46 (among the 119) patients who initially were ThyAb negative, became ThyAb positive after a mean of 6.2 years; one had an elevated TSH (21). Among 37 SLE patients, positive anti-thyroperoxidase antibodies (AbTPO) were found in 11 (61%) of 18 patients with AbM, and in 3 (16%)/19 without (22). In 12 children with active SLE, aged 5–18 years, AbTg were positive in 7/12. The serum levels of triiodothyronine (T3), T4, and TSH were in the reference limits in all SLE children (23). Forty-five Egyptian SLE patients (43 women and 2 men) were evaluated for thyroid disorders, versus a group of 20 normal females. The mean serum free T3 levels in all patients were significantly lower than in controls (1.89 ± 1.14 versus 3.15 ± 0.93 pg/ml; *P* < 0.05), overall in patients treated with cyclophosphamide. Also serum free T4 levels in SLE patients were significantly lower than in controls. Two of 45 patients (4.4%) had primary hypothyroidism. The mean serum TSH levels in SLE patients were significantly higher than in controls (4.82 ± 22.2 versus 2.65 ± 1.18 μIU/ml; *P* < 0.001). Among six patients with decreased TSH levels, one showed decreased T3 and T4, two had decreased T4 only (24). In another study, the prevalences of HT, or Graves’ disease (GD) in SLE patients, were 90-fold, and 68-fold, higher than in the general population (25).

Autoimmune thyroid disorders were evaluated in families with more than one SLE patient. Among 1,138 SLE patients, 169 had a diagnosis of Sjögren’s syndrome (SS), of whom 50 (29.6%) also had AITD. Among the 939 patients with SLE without SS, 119 (12.7%) had AITD. Among 2,291 SLE-unaffected relatives, 44 had diagnosed primary SS and 16 (36.3%) of these also had autoimmune thyroid disease. 265/2,247 (11.8%) subjects had autoimmune thyroid disease. These findings suggested that autoimmune thyroid disease is observed in excess among SLE patients with a diagnosis of secondary SS, as among their SLE-unaffected relatives with a diagnosis of primary SS (26). Seventy-seven SLE patients were studied for thyroid disorders, against 52 controls. Hypothyroidism was reported in 11.6% of SLE patients compared to 1.9% of controls. None of the patients or controls had hyperthyroidism. No statistically significant difference was observed in the levels of anti-thyroglobulin antibodies (AbTg) or AbTPO between the study group and the control group. No association was found between the SLE disease activity (SLEDAI) score and the prevalence of ThyAb (27). In 524 patients with SLE, AITD were evaluated, versus 50 female adults. 32/524 (6.1%) SLE patients and 1/50 controls had symptomatic autoimmune thyroid dyfunctions, in particular hypothyroidism (28 SLE patients versus 1 control). Sixty (11.5%) SLE patients had subclinical thyroid diseases and 89/524 (17%) had positive ThyAb in absence of thyroid dysfunctions. ThyAb forerun the appearance of clinical autoimmune thyroid disease in 70% of SLE patients. SS and positive rheumatoid factor were more frequent in SLE patients with AITD than in those without. SLEDAI was correlated with the presence of hyperthyroidism (28). The prevalence of AITD in 80 patients with SLE or RA was significantly higher than in the 34 controls (24 versus 8%, *P* < 0.05) (29). Two hundred thirteen SLE patients were evaluated by assessing thyroid hormones, the presence of ThyAb, and thyroid ultrasonography with respect to 426 controls (matched by age and gender), from the same geographic area, with a well-defined status of iodine intake. In female SLE patients versus controls, the odds ratio (OR) was 4.5 [95% confidence interval (CI), 2.5–8.4] for subclinical hypothyroidism; 2.9 (95% CI, 2.0–4.4) for thyroid autoimmunity; and 2.6 (95% CI, 1.7–4.1) for AbTPO positivity. Female SLE patients had significantly (*P* < 0.01) higher mean values of TSH and AbTPO than controls, as a significantly (*P* < 0.01) higher prevalence of clinical hypothyroidism and GD. In this study, 3% of SLE patients had “non-thyroidal illness syndrome” versus 0 controls (7). Hundred SLE patients were also evaluated for AITD, in comparison with 100 controls (matched by sex and age). Thyroid dysfunction was reported in 36 (36%) (all women) SLE patients [14 (14%) with clinical hypothyroidism, 2 (2%) with subclinical hyperthyroidism, and 12 (12%) with subclinical hypothyroidism], versus 8 (8%) of controls. Eight patients (8%) had isolated low T3 in agreement with ESS. Eighteen (50%) of thyroid dysfunctions were of autoimmune origin with positive autoantibodies, but not the other 18 (50%). Twelve (12%) of SLE patients had elevated ThyAb alone, while only five (5%) of controls had primary hypothyroidism and three (3%) had subclinical hypothyroidism, and no cases of hyperthyroidism were reported. SLEDAI and thyroid dysfunction of sick euthyroid type were significantly associated. Prevalence of ThyAb in SLE patients was 30% with respect to 10% of controls. There were no other autoimmune endocrine diseases such as diabetes or Addison’s disease in SLE patients (30). Among a total of 63 pregnant SLE women, 13% were on thyroid hormone prior to becoming pregnant, 11% were diagnosed with hypothyroidism during pregnancy, and 14% developed postpartum thyroiditis. The prevalence of preterm delivery was 67% in women with thyroid disease and 18% in women who were thyroid disease free. The presence of ThyAb was not correlated with preterm delivery. This study suggests that pregnant women with SLE have an increased prevalence of thyroid disease. Women with SLE and thyroid disease have an increased prevalence of preterm delivery (31). The prevalence of thyroid diseases was retrospectively analyzed in 1,006 Chinese SLE patients. The prevalence of AITD was 2.78%, central hypothyroidism 1.29%, clinical hypothyroidism 1.69%, subclinical hypothyroidism 10.04%, hyperthyroidism 1.19%, ESS 9.54%, and nodules 1.09%, respectively. Subclinical hypothyroidism was more prevalent (10.04%) in this study, than the prevalence of thyroid abnormalities in the general Chinese population (0.91–6.05%). Moreover, patients with lupus nephritis had subclinical hypothyroidism more frequently (13.4%) than those without (7.3%, *P* = 0.001) (32).

However, discordant results have been recently reported (33). In 1,633 SLE patients of new diagnosis, the prevalence of hyperthyroidism, hypothyroidism, and autoimmune thyroiditis (AT) was compared with 6,532 controls (matched by sex and age). The cumulative incidence of thyroid disease in SLE patients was lower than in control subjects (8.1 versus 16.9%, *P* < 0.001). The authors suggested that SLE patients had a significantly lower rate of thyroid diseases and hyperthyroidism than matched controls (33). The reported study could have some limitations, able to affect the results, to be considered: (1) it was based on diagnostic codes released from The National Health Insurance Research Database, and for this reason details on thyroid serological assessments, presence of ThyAb, or SLE autoantibodies were not available; (2) data obtained in a retrospective cohort study are usually inferior in statistical quality to those derived from randomized trials because of the potential biases related to adjustments for confounding variables (33). A total of 376 Colombian SLE patients were evaluated for the presence of (1) confirmed autoimmune hypothyroidism (AH), (2) positive AbTPO/AbTg without hypothyroidism, (3) non-AH, and (4) SLE patients with neither. Confirmed AH prevalence was 12%. AbTg and AbTPO were reported in 10% and 21% euthyroid SLE patients, respectively. Patients with confirmed AH were significantly older and had later age at the onset of the disease. SS [adjusted OR (AOR) 23.2, 95% CI, 1.89–359.53, *P* = 0.015], smoking (AOR 6.93, 95% CI, 1.98–28.54, *P* = 0.004), and positive anticyclic citrullinated peptide (AOR 10.35, 95% CI, 1.04–121.26, *P* = 0.047) were associated with AH in SLE patients. Female gender, smoking, older age, SS, certain autoantibodies, and articular and cutaneous involvement were associated with this polyautoimmunity (34). Another meta-analysis evaluated the association of SLE and thyroid autoimmunity; a total of 1,076 SLE cases and 1,661 healthy controls were included. The meta-analysis results showed that the prevalence of ThyAb positivity in SLE patients was higher than in healthy controls (AbTg: OR = 2.99, 95% CI, 1.83–4.89; AbTPO: OR = 2.20, 95% CI, 1.27–3.82, respectively) (35). The frequency of AITD among 189 SLE patients was 6.3%, with 2.6% in the hyperthyroid group and 3.7% in the hypothyroid group. A new association between AITD and antiphospholipid syndrome was shown (36). Five thousand and eighteen patients with SLE and 25,090 age- and sex-matched controls were evaluated for thyroid dyfunctions. The proportion of hypothyroidism in SLE patients was increased with respect to the prevalence in controls (15.58 and 5.75%, respectively, *P* < 0.001). In a multivariate analysis, SLE was associated with hypothyroidism (OR = 2.644, 95% CI, 2.405–2.908) (37). Conversely, a recent study evaluated prospectively the prevalence of other autoimmune disorders in outpatient clinic in 3,069 consecutive patients with diagnosed chronic AT, versus two age- and sex-matched control groups: (a) a control group of 1,023 subjects, drawn from a random sample of the general population without thyroid disorders and (b) 1,023 patients with non-toxic multinodular goiter from the same random sample of the general population, with similar iodine intake. The results of the study demonstrated a significant increase of SLE prevalence in AT patients (versus both controls) (38).

### Th1 and Th2 Cytokines in SLE

The ratios of Th1 and Th2 cytokines have been investigated to determine the cytokine homeostasis in SLE. Even if SLE was thought to be a Th2-polarized disease (39), more recently significantly elevated circulating cytokines of Th1 response, including TNF-α, and IFN-γ were also shown in SLE patients (40–42). Chemokine IFN-γ-inducible protein 10, the prototype of the chemokine (C-X-C motif) family, has chemotactic activity especially for activated Th1 cells and is involved in the pathogenesis of various Th1-dominant autoimmune diseases (43), and in SLE. Also AITD are Th1 immune-mediated autoimmune disorders in which Th1 lymphocytes, IFN-γ, and IFN-γ-dependent chemokines (CXCL9, CXCL10, CXCL11) play an important role (44–46). The common Th1 immune predominance in AITD and SLE should be the immunopathogenetic base of the association of these two diseases. On the whole, the abovementioned results show a high prevalence, and incidence, of new cases of hypothyroidism and AT in SLE patients, and suggest that female patients with systemic sclerosis, who are at high risk [a borderline high (even if in the normal range) TSH value, positive AbTPO, and a hypoechoic and small thyroid] should have periodic thyroid function follow-up.

## Thyroid Abnormalities and Clinical Aspects of SLE

Many studies have tried to associate thyroid abnormalities with clinical findings of SLE, with different results.

An association between hypothyroidism, or AITD, and SLE clinical activity has been described in few studies (18, 28).

While another study found an association between SLEDAI score and ESS (30).

The reported data suggested that AITD is found more frequently among patients with SLE with a diagnosis of secondary SS, but not in SLE patients without SS (26, 28).

However most of the studies were not able to find any association among thyroid autoimmunity or thyroid dysfuntions, and clinical or serological features of SLE.

## SLE and Papillary Thyroid Cancer (PTC)

A first prospective study investigated the prevalence and features of thyroid cancer in 153 unselected SLE patients in comparison with two population-based, control groups (matched by sex and age): (1) 459 iodine-deficient controls and (2) 459 iodine-sufficient controls. SLE patients had circulating TSH, AbTg and AbTPO levels significantly higher (*P* < 0.001 for all), and a higher prevalence of hypothyroidism (*P* < 0.001), than controls. Five PTC cases were reported in SLE patients, none in iodine-deficient controls (*P* = 0.001), and only one was shown in iodine-sufficient controls (*P* = 0.001). Thyroid autoimmunity was shown in 80% of SLE patients with confirmed thyroid cancer, and only in 31% of SLE patients without thyroid cancer (*P* = 0.02). These findings suggested that PTC prevalence in SLE patients is more elevated than in controls, especially in the presence of thyroid autoimmunity (8). Another study showed that among 16,409 patients [121,283 (average 7.4) person-years], 644 cases of cancer occurred, in particular hematologic ones, and a raised risk of thyroid cancer (SIR 1.76, 95% CI, 1.13–2.61) was observed too (47). A systematic review with meta-analysis investigated the risk of thyroid cancer in SLE revealing the positive association between thyroid cancer and SLE risk (48). A further meta-analysis assessed the association of SLE and malignancy evaluating 16 papers, including 59,662 SLE patients. The pooled relative risks were 1.28 (95% CI, 1.17–1.41) for overall cancer, 1.78 (95% CI, 1.35–2.33) for thyroid cancer (49). Also other studies confirmed the association between SLE and thyroid cancer (50).

## Conclusion

Most of the studies show high prevalence, and incidence, of new cases of hypothyroidism and AT in SLE patients, overall in female gender. A limited number of GD cases have been also reported in SLE, in agreement with the higher prevalence of thyroid autoimmunity. It has been also demonstrated that Th1 predominance is associated with AT in SLE patients.

Furthermore, a higher prevalence of PTC has been recently reported in SLE patients, overall in the presence of thyroid autoimmunity. However, studies in larger number of SLE patients are needed to confirm data about thyroid cancer.

On the whole, data from literature strongly suggest that female SLE patients, with a high risk (a normal but at the higher limit TSH value, positive AbTPO, a hypoechoic pattern, and small thyroid) should undergo periodic thyroid function follow-up, and appropriate treatments when needed. However, studies in larger number of patients are required to evaluate if routine thyroid screening could be beneficial for SLE patients.

A careful thyroid monitoring would be opportune during the follow-up of these patients.

## Author Contributions

SMF, GE, CV, MC, AA, and PF gave substantial contribution in the conception and design of the work, and in writing the paper; gave the final approval of the version to be published; agreed to be accountable for all aspects of the work in ensuring that questions related to the accuracy or integrity of any part of the work are appropriately investigated and resolved. MC, AA, and PF revised it critically for important intellectual content.

## Conflict of Interest Statement

The authors declare that the research was conducted in the absence of any commercial or financial relationships that could be construed as a potential conflict of interest.

## Abbreviations

AbM, antimicrosomal antibodies; AbTg, anti-thyroglobulin antibodies; AbTPO, antithyroid peroxidase antibodies; AH, autoimmune hypothyroidism; AITD, autoimmune thyroid disorders; AOR, adjusted odds ratio; APS, antiphospholipid syndrome; AT, autoimmune thyroiditis; ESS, euthyroid sick syndrome; GD, Graves’ disease; IFN, interferon; IL, interleukin; OR, odds ratio; PTC, papillary thyroid cancer; RA, rheumatoid arthritis; SLE, systemic lupus erythematosus; SLEDAI, SLE disease activity; SS, Sjögren’s syndrome; T4, thyroxine; T3, triiodothyronine; TBII, TSH-binding inhibitor immunoglobulin; ThyAb, thyroid autoantibodies; TNF, tumor necrosis factor; TSH, thyroid-stimulating hormone; TSI, thyroid-stimulating immunoglobulins.

## References

[1] Rahman A Isenberg DA Systemic lupus erythematosus. N Engl J Med (2008) 358:929–39. 10.1056/NEJMra071297 18305268

[2] Lisnevskaia L Murphy G Isenberg D. Systemic lupus erythematosus. Lancet (2014) 384:1878–88. 10.1016/S0140-6736(14)60128-8 24881804

[3] Jeltsch-David H Muller S Neuropsychiatric systemic lupus erythematosus and cognitive dysfunction: the MRL-lpr mouse strain as a model. Autoimmun Rev (2014) 13:963–73. 10.1016/j.autrev.2014.08.015 25183233

[4] Funauchi M Ikoma S Enomoto H Horiuchi A. Decreased Th1-like and increased Th2-like cells in systemic lupus erythematosus. Scand J Rheumatol (1998) 27:219–24. 10.1080/030097498440859 9645418

[5] Prud’homme GJ Kono DH Theofilopoulos AN. Quantitative polymerase chain reaction analysis reveals marked overexpression of interleukin-1 beta, interleukin-1 and interferon-gamma mRNA in the lymph nodes of lupus-prone mice. Mol Immunol (1995) 32:495–503. 10.1016/0161-5890(95)00024-9 7783752

[6] Mak A Kow NY. The pathology of T cells in systemic lupus erythematosus. J Immunol Res (2014) 2014:419029. 10.1155/2014/419029 24864268 PMC4017881

[7] Antonelli A Fallahi P Mosca M Ferrari SM Ruffilli I Corti A Prevalence of thyroid dysfunctions in systemic lupus erythematosus. Metabolism (2010) 59:896–900. 10.1016/j.metabol.2009.10.010 20005534

[8] Antonelli A Mosca M Fallahi P Neri R Ferrari SM D’Ascanio A Thyroid cancer in systemic lupus erythematosus: a case-control study. J Clin Endocrinol Metab (2010) 95:314–8. 10.1210/jc.2009-0677 19906791

[9] Hijmans W Doniach D Roitt IM Holborow EJ Serological overlap between lupus erythematosus, rheumatoid arthritis, and thyroid auto-immune disease. Br Med J (1961) 2:909–14. 10.1136/bmj.2.5257.909 13714204 PMC1969944

[10] Halberg P Bertram U Soborg M Nerup J Organ antibodies in disseminated lupus erythematosus. Acta Med Scand (1965) 178:291–9. 10.1111/j.0954-6820.1965.tb04273.x 5318558

[11] Jonsson H Nived O Sturfelt G Thyroid disorders in systemic lupus erythematosus are associated with secondary Sjögren’s syndrome. Ann Rheum Dis (1987) 46:349 10.1136/ard.46.4.349-a PMC1002134 3592794

[12] Sakata S Nakamura S Nagai K Komaki T Kawade M Niwa T Two cases of systemic lupus erythematosus associated with hyperthyroidism. Jpn J Med (1987) 26:373–6. 10.2169/internalmedicine1962.26.373 3694920

[13] Goh KL Wang F. Thyroid disorders in systemic lupus erythematosus. Ann Rheum Dis (1986) 45:579–83. 10.1136/ard.45.7.579 3740982 PMC1001940

[14] Weetman AP Walport MJ. The association of autoimmune thyroiditis with systemic lupus erythematosus. Br J Rheumatol (1987) 26:359–61. 10.1093/rheumatology/26.5.359 3664160

[15] Miller FW Moore GF Weintraub BD Steinberg AD. Prevalence of thyroid disease and abnormal thyroid function test results in patients with systemic lupus erythematosus. Arthritis Rheum (1987) 30:1124–31. 10.1002/art.1780301006 3675658

[16] Baker JR Jr Miller FW Steinberg AD Burman KD. Thyroid stimulating and thyrotrophin binding-inhibitory immunoglobulin activity in patients with systemic lupus erythematosus having thyroid function abnormalities. Thyroid (1991) 1:229–34. 10.1089/thy.1991.1.229 1688101

[17] Vianna JL Haga HJ Asherson RA Swana G Hughes GR A prospective evaluation of antithyroid antibody prevalence in 100 patients with systemic lupus erythematosus. J Rheumatol (1991) 18:1193–5. 1941823

[18] Magaro M Zoli A Altomonte L Mirone L La Sala L Barini A The association of silent thyroiditis with active systemic lupus erythematosus. Clin Exp Rheumatol (1992) 10:67–70. 1551281

[19] Tsai RT Chang TC Wang CR Chuang CY Chen CY. Thyroid disorders in Chinese patients with systemic lupus erythematosus. Rheumatol Int (1993) 13:9–13. 10.1007/BF00290328 8516625

[20] Boey ML Fong PH Lee JS Ng WY Thai AC Autoimmune thyroid disorders in SLE in Singapore. Lupus (1993) 2:51–4. 10.1177/096120339300200109 8485560

[21] Kausman D Isenberg DA. Thyroid autoimmunity in systemic lupus erythematosus: the clinical significance of a fluctuating course. Br J Rheumatol (1995) 34:361–4. 10.1093/rheumatology/34.4.361 7788152

[22] Tsai RT Chang TC Wang CR Lee SL Wang CJ Tsay GJ. Thyroid peroxidase autoantibodies and their effects on enzyme activity in patients with systemic lupus erythematosus. Lupus (1995) 4:280–5. 10.1177/096120339500400408 8528224

[23] Mihailova D Grigorova R Vassileva B Mladenova G Ivanova N Stephanov S Autoimmune thyroid disorders in juvenile chronic arthritis and systemic lupus erythematosus. Adv Exp Med Biol (1999) 455:55–60. 10.1007/978-1-4615-4857-7_8 10599323

[24] Shahin AA Mostafa H Mahmoud S. Thyroid hormones and thyroid-stimulating hormone in Egyptian patients with systemic lupus erythematosus: correlation between secondary hypothyroidism and neuropsychiatric systemic lupus erythematosus syndromes. Mod Rheumatol (2002) 12:338–41. 10.3109/s101650200060 24384003

[25] Biró E Szekanecz Z Czirják L Dankó K Kiss E Szabó NA Association of systemic and thyroid autoimmune diseases. Clin Rheumatol (2006) 25:240–5. 10.1007/s10067-005-1165-y 16247581

[26] Scofield RH Bruner GR Harley JB Namjou B Autoimmune thyroid disease is associated with a diagnosis of secondary Sjögren’s syndrome in familial systemic lupus. Ann Rheum Dis (2007) 66:410–3. 10.1136/ard.2006.055103 16984944 PMC1856020

[27] Mader R Mishail S Adawi M Lavi I Luboshitzky R. Thyroid dysfunction in patients with systemic lupus erythematosus (SLE): relation to disease activity. Clin Rheumatol (2007) 26:1891–4. 10.1007/s10067-007-0602-5 17372671

[28] Appenzeller S Pallone AT Natalin RA Costallat LT. Prevalence of thyroid dysfunction in systemic lupus erythematosus. J Clin Rheumatol (2009) 15:117–9. 10.1097/RHU.0b013e31819dbe4c 19300286

[29] Lazúrová I Benhatchi K Rovenský J Kozáková D Wagnerová H Tajtáková M Autoimmune thyroid disease and autoimmune rheumatic disorders: a two-sided analysis. Ann N Y Acad Sci (2009) 1173:211–6. 10.1111/j.1749-6632.2009.04809.x 19758153

[30] Kumar K Kole AK Karmakar PS Ghosh A. The spectrum of thyroid disorders in systemic lupus erythematosus. Rheumatol Int (2012) 32:73–8. 10.1007/s00296-010-1556-5 20658291

[31] Stagnaro-Green A Akhter E Yim C Davies TF Magder L Petri M. Thyroid disease in pregnant women with systemic lupus erythematosus: increased preterm delivery. Lupus (2011) 20:690–9. 10.1177/0961203310394894 21436215

[32] Gao H Li C Mu R Guo YQ Liu T Chen S Subclinical hypothyroidism and its association with lupus nephritis: a case control study in a large cohort of Chinese systemic lupus erythematosus patients. Lupus (2011) 20:1035–41. 10.1177/0961203311401456 21646314

[33] Lin WY Chang CL Fu LS Lin CH Lin HK. Systemic lupus erythematosus and thyroid disease: a 10-year study. J Microbiol Immunol Infect (2015) 48:676–83. 10.1016/j.jmii.2014.03.004 24874431

[34] Franco JS Amaya-Amaya J Molano-González N Caro-Moreno J Rodríguez-Jiménez M Acosta-Ampudia Y Autoimmune thyroid disease in Colombian patients with systemic lupus erythematosus. Clin Endocrinol (Oxf) (2015) 83:943–50. 10.1111/cen.12662 25382266

[35] Pan XF Gu JQ Shan ZY. Patients with systemic lupus erythematosus have higher prevalence of thyroid autoantibodies: a systematic review and meta-analysis. PLoS One (2015) 10:e0123291. 10.1371/journal.pone.0123291 25905898 PMC4408090

[36] Ong SG Choy CH. Autoimmune thyroid disease in a cohort of Malaysian SLE patients: frequency, clinical and immunological associations. Lupus (2016) 25:67–74. 10.1177/0961203315593164 26113361

[37] Watad A Mahroum N Whitby A Gertel S Comaneshter D Cohen AD Hypothyroidism among SLE patients: case-control study. Autoimmun Rev (2016) 15:484–6. 10.1016/j.autrev.2016.01.019 26826435

[38] Fallahi P Ferrari SM Ruffilli I Elia G Biricotti M Vita R The association of other autoimmune diseases in patients with autoimmune thyroiditis: review of the literature and report of a large series of patients. Autoimmun Rev (2016) 15:1125–8. 10.1016/j.autrev.2016.09.009 27639841

[39] Mohan C Adams S Stanik V Datta SK. Nucleosome: a major immunogen for pathogenic autoantibody-inducing T cells of lupus. J Exp Med (1993) 177:1367–81. 10.1084/jem.177.5.1367 8478612 PMC2191002

[40] Davas EM Tsirogianni A Kappou I Karamitsos D Economidou I Dantis PC. Serum IL-6, TNFalpha, p55 srTNFalpha, p75srTNFalpha, srIL-2alpha levels and disease activity in systemic lupus erythematosus. Clin Rheumatol (1999) 18:17–22. 10.1007/s100670050045 10088943

[41] Al-Janadi M Al-Balla S Al-Dalaan A Raziuddin S. Cytokine profile in systemic lupus erythematosus, rheumatoid arthritis, and other rheumatic diseases. J Clin Immunol (1993) 13:58–67. 10.1007/BF00920636 8445045

[42] Apostolidis SA Lieberman LA Kis-Toth K Crispín JC Tsokos GC. The dysregulation of cytokine networks in systemic lupus erythematosus. J Interferon Cytokine Res (2011) 31:769–79. 10.1089/jir.2011.0029 21877904 PMC3189553

[43] Zlotnik A Yoshie O Chemokines: a new classification system and their role in immunity. Immunity (2000) 12:121–7. 10.1016/S1074-7613(00)80165-X 10714678

[44] Antonelli A Ferrari SM Corrado A Di Domenicantonio A Fallahi P. Autoimmune thyroid disorders. Autoimmun Rev (2015) 14:174–80. 10.1016/j.autrev.2014.10.016 25461470

[45] Antonelli A Ferrari SM Giuggioli D Ferrannini E Ferri C Fallahi P. Chemokine (C-X-C motif) ligand (CXCL)10 in autoimmune diseases. Autoimmun Rev (2014) 13:272–80. 10.1016/j.autrev.2013.10.010 24189283

[46] Antonelli A Ferrari SM Frascerra S Pupilli C Mancusi C Metelli MR CXCL9 and CXCL11 chemokines modulation by peroxisome proliferator-activated receptor-alpha agonists secretion in Graves’ and normal thyrocytes. J Clin Endocrinol Metab (2010) 95:E413–20. 10.1210/jc.2010-0923 20810571

[47] Bernatsky S Ramsey-Goldman R Labrecque J Joseph L Boivin JF Petri M Cancer risk in systemic lupus: an updated international multi-centre cohort study. J Autoimmun (2013) 42:130–5. 10.1016/j.jaut.2012.12.009 23410586 PMC3646904

[48] Zhang M Li XM Wang GS Qian L Tao JH Ma Y Thyroid cancer in systemic lupus erythematosus: a meta analysis. Int J Clin Exp Pathol (2014) 7:6270–3. 25337279 PMC4203250

[49] Cao L Tong H Xu G Liu P Meng H Wang J Systemic lupus erythematous and malignancy risk: a meta-analysis. PLoS One (2015) 10:e0122964. 10.1371/journal.pone.0122964 25885411 PMC4401738

[50] Goobie GC Bernatsky S Ramsey-Goldman R Clarke AE. Malignancies in systemic lupus erythematosus: a 2015 update. Curr Opin Rheumatol (2015) 27:454–60. 10.1097/BOR.0000000000000202 26125105 PMC4562287

